# Beyond Fear of Crime: Exploring the True Worries of Older Adults in the Context of Fear of Crime and Vulnerability in Sweden

**DOI:** 10.1007/s10610-025-09631-2

**Published:** 2025-06-25

**Authors:** Maria Camacho Doyle, Frida Bood, Louise Frogner, Nadezhda Golovchanova, Karin Hellfeldt

**Affiliations:** School of Behavioral, Social and Legal Sciences, Örebro University, Fakultetsgatan 1, 701 82, 019 303000 Örebro, Sweden

**Keywords:** Relevance of fear, Fear of crime, Perceived risk, Avoidant behavior, Frailty, Older adults

## Abstract

The current study examines the relative importance of fear of crime and its three aspects—affective, behavioral, and cognitive—in relation to frailty (daily activities, health problems, psychosocial functioning), news consumption, trust in neighborhood, and perceptions of rising crime among older adults in a mid-sized Swedish municipality. Using logistic regression, we analyzed data from a subsample of 336 participants from a Swedish cross-sectional study of adults aged 65 and older (mean age = 76.62; age range = 64 to 106 years). Contrary to the common portrayal of older adults as highly fearful of crime, our findings reveal that their primary worries are more closely linked to health problems, social isolation, and other personal vulnerabilities than to fear of crime. Trust in the neighborhood is associated with lower levels of the affective and behavioral aspects of fear of crime. Conversely, perceptions of rising crime are positively related to the affective and behavioral aspects of fear. Health problems and problems in psychosocial functioning are positively linked to the affective aspect. Further, problems in psychosocial functioning are positively related to the behavioral aspect. Overall, while crime is a worry, it is overshadowed by more immediate health and social issues.

**Supplementary Information:**

The online version contains supplementary material available at https://doi.org/10.1007/s10610-025-09631-2.

## Introduction

Fear of crime (FoC) among older adults has been highlighted in research, media, and political discourse as a societal concern. Historically, studies have examined the so-called victimization-fear paradox, which suggests that older adults are more fearful of crime despite being less likely to be victimized (Hale, 1996). Recent studies challenge this paradox, indicating that older age alone does not fully account for FoC among older adults (Collins, 2016; Fisher et al., 2004; Kappes et al., 2013; Ziegler & Mitchell, 2003). One point of criticism has been that previous studies overlook the role of FoC within the broader aging context, which includes various themes, worries, and challenges that older adults’ face. This raises an important question: How significant is FoC compared to other life worries faced by older adults?

Research suggests that Fear of Crime (FoC) is a multi-leveled construct comprising affective (A; the fear itself, experienced as an emotion), behavioral (B; actions taken or refrained from when experiencing fear), and cognitive (C; perceived risk of becoming a victim of crime) aspects (Camacho Doyle et al., 2022; Cross & Lee, 2022; Gabriel & Greve, 2003; Greve, 1998; Greve et al., 2018; Guedes et al., 2018; Jackson & Gouseti, 2012; Kappes et al., 2013; Rader, 2004, 2017; Rader & Haynes, 2014; Rühs et al., 2017). Studies indicate that older adults are more fearful of crime at the behavioral level (B), often taking precautions, such as avoiding certain places or refraining from activities due to their FoC (Greve, 1998; Greve et al., 2018; Rühs et al., 2017). This aligns with research indicating that individuals typically respond to potential victimization threats in one of three ways: affectively (A), by feeling fear; behaviorally (B), by taking precautions; or cognitively (C), by assessing their risk (Rader & Haynes, 2014) and highlights the need to focus on these dimensions within older populations. While the relationships between the causes and consequences of FoC are not fully established (see Rader, 2017), these aspects may sometimes work together or predict one another (see Erčulj, 2022). Older individuals are particularly inclined to alter their behavior in response to their fears, emphasizing the significance of examining the behavioral aspect (B) of FoC in this demographic.

Beyond the complexity of FoC and its different aspects, various individual and contextual factors significantly relate to all aspects of FoC within the general population (Camacho Doyle et al., 2022). At the individual level (see e.g., Lane et al., 2014; May & Dunaway, 2000; Schafer et al., 2006; Rader et al., 2012), vulnerability—such as frailty—media consumption, and concerns about rising crime rates play crucial roles. At the contextual level (see e.g., Guldåker et al., 2024; Kuen et al., 2022; Kronkvist, 2022; Markowitz et al., 2001; O’Brien et al., 2019; Robinson et al., 2003; Wyant, 2008), a lack of social cohesion in neighborhoods can exacerbate feelings of loneliness and perceived risk. However, the relationships between various individual predictors (such as frailty and media consumption) and contextual factors (like social cohesion in neighborhoods) and their connection to the ABC aspects of FoC has not been comprehensively studied in older populations. This gap emphasizes the need for further research to understand how these factors relate to FoC and its importance among other worries in older adults.

Although studies have addressed FoC in advanced age (Golovchanova et al., 2021, 2022; Greve, 1998; Greve et al., 2018; Rühs et al., 2017), research on the relative importance of FoC for older people is relatively limited. Greve (1998) showed that FoC was not considered a big problem when compared to other life worries in a sample of older adults. Accidents, illness, becoming dependent, losing a friend or a relative, worry about retirement and healthcare, as well as environmental worries all scored higher than FoC as possible threats. These results are generally in line with the worries and fears typical for advanced age as a life stage outlined in gerontological literature (see e.g., Kuin & Spaans, 2017; Lenze & Wetherell, 2011). Specifically, fears commonly experienced by older adults include fear of dementia, fear of sickness or death of a loved one, fear of own premature death, fear to lose autonomy and independence, fear of fall accident, and other existential fears. When asked in an open question format, older adults mention potential violence, illness, loneliness, growing older and dying, and other factors among significant reasons for fear (Jakobsson and Hallberg, 2005). But research on the relative importance of FoC among other significant life worries and stressors for older adults is limited. Even less is known about the individual and contextual factors that may influence the degree to which FoC is important for older adults. The current study aims to address these knowledge gaps by exploring the relative importance of FoC for older adults among other life worries and to investigate the associations between individual and contextual factors with such relative importance of FoC and with the different aspects of FoC in an older population.

### Theoretical Explanations for Fear of Crime

When explaining FoC, theoretical explanations of individual and contextual levels have been considered. Research on mechanisms at the individual level (see e.g., Lane et al., 2014; May & Dunaway, 2000; Schafer et al., 2006; Rader et al., 2012), and the contextual level (see e.g., Guldåker et al., 2024; Kuen et al., 2022; Kronkvist, 2022; Markowitz et al., 2001; O’Brien et al., 2019; Robinson et al., 2003; Wyant, 2008) has mainly focused on the general population. How these factors relate to FoC among older adults is less studied.

Earlier research suggests that differences in perceived safety could be due to individual differences in susceptibility to fear (Gabriel & Greve, 2003; Jackson, 2009, 2015). According to the *vulnerability perspective*, important individual level factors associated with FoC are older age, female gender, and diminished health (see Rader, 2017). Thus, older people may be more vulnerable to fear because their reduced physical resistance could lower their perceived ability to defend themselves in a threatening situation (Henson & Reyns, 2015; Killias, 1990). Additionally, several studies have shown that older women tend to report more FoC than older men (e.g., Acierno et al., 2004; Bazargan, 1994; BRÅ, 2018; De Donder et al., 2012; Hanslmaier et al., 2018; Olofsson et al., 2012; Stafford et al., 2007). Finally, diminished health has been associated with increased FoC in older adults (Bazargan, 1994; Hanslmaier et al., 2018; Olofsson et al., 2012).

Such individual level vulnerability explanations are in line with both *rationalist paradigm* and *symbolic paradigms* which are especially applicable to understanding the nature of FoC in late life (De Donder et al., 2009; Elchardus et al., 2008). The rationalist paradigm suggests that individuals are generally rational in their estimation of risks (De Donder et al., 2009). For instance, older people with less physical strength and thus less chance of withstanding a perpetrator are expected to be more fearful. According to the rationalist paradigm, older people are expected to display levels of FoC that are adequate to perceived risks of victimization (De Donder et al., 2009; Elchardus et al., 2008). However, an alternative *symbolic paradigm* postulates that vulnerability can stem from various reasons above and beyond factors associated with threats of crime. For instance, ill-health or being an object of ageism might result in general feelings of vulnerability and unsafety, which in turn manifest as FoC. Taken together, both rationalist and symbolic paradigms underscore vulnerability in its various aspects, thus making individual factors, such as older age, female gender, and diminished health, important of understanding FoC.

An alternative explanation of FoC is provided by a range of contextual factors. According to the"*fear of crime loop*"concept (see e.g., Altheide, 2002, 2006; Heber, 2009), FoC is influenced by how prominently the issue is discussed in society. Factors like media portrayals of crime and political debates can significantly shape individuals’ fears. For instance, research shows that vicarious victimization—knowing someone who has been a crime victim or hearing about such incidents in the media—can increase an individual's FoC (Chiricos et al., 2000; Eschholz et al., 2003; Skogan & Maxfield, 1981). Various forms of media consumption have previously been linked to increased FoC (Eschholz et al., 2003; Hollis et al., 2017; Intravia et al., 2017; Kohm et al., 2012; Näsi et al., 2021; Roche et al., 2016; Smolej, 2011). This suggests that media consumption might relate to FoC.

An early model of FoC suggests that fear is a rational response to the perceived risk of victimization (see e.g., Farrall et al., 2007; Greve et al., 2018; Hale, 1996). This fear is rooted in beliefs about the likelihood of becoming a victim, influenced by actual crime rates (Doran & Burgess, 2012; Hale, 1996; May & Dunaway, 2000; Schafer et al., 2006) and information about crime rates. Higher crime rates in neighborhoods or recent reports of criminal activity can increase fear (Brantingham & Brantingham, 1995) as individuals interpret these indicators as signs of rising societal crime. Perceived safety has been associated with crime levels and public perceptions of criminal activity (Brunton-Smith & Sturgis, 2011; Doran & Burgess, 2012; Hale, 1996; Jackson, 2006). Previous crime also contributes potentially to why individuals may experience FoC (Luo et al., 2016; Rader, et al., 2012; Wilcox-Rountree and Land, 1996a, 1996b; Zhao et al., 2015).

Another contextual factor possibly linked to FoC is social cohesion and trust in other people in the neighborhood (see e.g., Sampson et al., 1997). Collective efficacy is typically measured by combining perceived social trust (cohesion) and informal social control (Sampson, 2012). Social trust is vital, as it fosters informal control and encourages intervention for the common good. Communities with strong social trust are more resilient to crime and FoC. Residents who perceive higher social trust tend to feel more secure, as they see fewer problems in their community. High collective efficacy, driven by social cohesion and strong neighborly ties, is related to different aspects of FoC (Abdullah et al., 2015; Brunton-Smith & Sturgis, 2011; Brunton-Smith et al., 2014; Camacho Doyle et al., 2022; Hinkle, 2015; Markowitz et al., 2001; Swatt et al., 2013). Conversely, weak community ties are associated with higher fear (Camacho Doyle et al., 2022; Markowitz et al., 2001; Scarborough et al., 2010; Sampson et al., 1997). Thus, areas with strong social cohesion, a collective community, and low anonymity generally foster a greater sense of safety (see, e.g., Farrall et al., 2009).

### The Current Study

The association of individual and contextual factors and FoC has been extensively studied (see Henson & Reyns, 2015 for a review), but whether and how these factors differ for older adults remains relatively underexplored. While older adults may exhibit greater behavioral (B) FoC, they might not experience heightened affective (A) or cognitive (C) fear (Greve, 1998; Greve et al., 2018; Rühs et al., 2017). This underscores the need to differentiate between the ABC aspects of fear in advanced age and their potential explanatory factors, as FoC aspects might be differentially associated with individual and contextual factors relevant for older adults.

Also, previous research often overlooks how FoC fits within the broader context of aging and other significant life worries. How important is FoC compared to other late-life worries, such as health issues and loss of autonomy? It is important to study the older population specifically, rather than treating them as part of the general population, because understanding their specific fears allows for better-targeted interventions and support systems that enhance their sense of safety. Findings from studies focusing on older adults can inform policymakers and community organizations in developing age-appropriate safety programs and resources, ultimately creating environments that enhance the well-being of older individuals and reduce their fears.

The current study addresses the gap in research by examining how older adults rank FoC in relation to other common late life worries, such as health issues, physical weakness, and the illness or death of close ones. Additionally, we sought to examine how individual factors (e.g., frailty dimensions) and contextual factors (e.g., trust) are associated with the three aspects (ABC) of FoC.

The following questions were posed:

i) How do older adults rank FoC compared to other common fears and worries in late life? Based on gerontology literature, we anticipated that concerns related to personal health and autonomy would be ranked as more important than fear of crime.
ii) Which individual and contextual factors are independently associated with different aspects of FoC among older adults? Based on the vulnerability perspective, we expected that higher age, female gender, and greater frailty would contribute to fear of crime. In line with the rationalist paradigm, we anticipated that frailty in daily activities and health problems would have a stronger influence on fear of crime, whereas the symbolic paradigm suggests that psychosocial frailty might be a more significant contributing factor. Additionally, guided by the ‘fear of crime loop’, the rationalist paradigm, and evidence on social cohesion, we hypothesized that lower trust in neighbors, stronger beliefs that crime is increasing, and higher media consumption would be positively associated with fear of crime in older adults.

Since previous research (Greve et al., 2018; Kappes et al., 2013) has shown that older adults tend to report higher levels of behavioral fear of crime compared to its affective and cognitive components, we tentatively examined whether all theoretically relevant predictors would be more strongly associated with behavioral fear. In addition to exploring the determinants of different aspects of FoC, we also investigated the relationships between the perceived importance of FoC and various individual and contextual factors. Specifically, we analyzed the correlations between the importance assigned to FoC and variables such as trust in neighbors, belief in crime increase, and media consumption.

To summarize, this study offers a valuable methodological contribution to understanding fear of crime in the context of aging by applying the rank order measure that positions fear of crime among other common worries. This approach is relatively novel in the field, as most research has concentrated on the prevalence of fear of crime rather than its relative importance among worries in later life. Additionally, by focusing on older adults and age-relevant predictors, the study aims to inform policy by identifying the most suitable individual and contextual factors for targeted interventions to promote safety and well-being among older populations.

## Method

### Procedure

The current study used data from the 65 + and Safe Study (see Golovchanova et al., 2022), collected in 2019 in a mid-sized Swedish municipality. The survey was cross-sectional and focused on general safety and experiences of FoC among older adults living in senior apartments. Participants were eligible for the study if they (1) were 65 years or older in 2019, (2) were residents in a senior apartment managed by a Swedish municipal housing company, and (3) did not have severe cognitive impairment at the time. These senior apartments were regular rentals available to residents aged 65 years and above. Thus, the sample did not include for instance homeowners or nursing home residents. The senior apartments were equipped with specific design elements (e.g., elevators, automatic door openers), varied in sizes and were located across various urban and rural neighborhoods throughout the municipality.

The target sample for this data collection was determined by the Swedish Population Register made available by the Swedish Tax Agency. Examining the list of registered residents revealed that 1299 older adults met the study’s inclusion criteria. Further, residents who had moved or were deceased by the time of the data collection were excluded from the target sample, reducing it to 1256 individuals. A paper-and-pencil questionnaire, along with an information letter detailing the study, contact information for the research team, key ethical considerations, and instructions for completing either the paper or online version of the questionnaire, was sent to participants who met the inclusion criteria. The 65 + and Safe Study included 622 participants (49.5% of the final target sample). Among these, 587 participants completed the paper-questionnaire, and 35 participants completed the web-questionnaire; two participants completed both paper- and web- questionnaires, of whom only web responses were processed),

In this sample, 60.6% were women and the mean age was 77.6 (age range 64–106), 68% reported education level of a high school level or lower, and 56.6% lived alone. The project gained approval from the Swedish Ethical Review Agency in 2019 (id 2019–02248).

### Participants

The current study used a subsample from the 65 + and Safe study, including only participants with valid ratings for the item 'relative importance of FoC'. Thus, the subsample consisted of 336 participants, representing 54 percent of the 65 + and Safe Study sample. Of these, 57.1 percent were women, mean age 76.62 years, and age range of 64 to 106 years. In this subsample, 63.2% reported having education equivalent to a high school degree or lower, 34.6% were married, 5.7% lived together with a partner, 2.1% lived separate from a partner, 16.8% were divorced, 9.3% were single, and 31.4% were widowed. Further, 22.8% received assistance with daily tasks at home (e.g., with grocery shopping, household tasks, etc.), and 5.4% reported being a victim of a crime during the past 12 months. The subsample did not significantly differ from the full sample in terms of gender, χ^2^(2, *N* = 622) = 3.68, *p* = 0.55 but were slightly younger (*M* = 76.62, *SD* = 7.05) compared to the full sample (*M* = 78.75, *SD* = 7.26), *t*(620) = 3.70, *p* < 0.001.

### Measures

The current study aimed to assess FoC in four ways, the relative importance of FoC compared to other fears and the three aspects of FoC —affective, behavioral, and cognitive (ABC). However, the cognitive aspect (C) of FoC was not included in the logistic regression due to data-related issues. Specifically, the C component was excluded from the analysis due to a high proportion of missing data, only 84 out of 336 respondents (approximately 25%) provided responses to questions related to this aspect. This substantial number of missing values raised concerns regarding the reliability and generalizability of results associated with this variable. To maintain the integrity of the analysis and ensure valid conclusions, the exclusion of this component was deemed necessary. Additionally, participants assessed six potentially influencing factors as well as age and gender. Male was coded as 1 and female as 2. Age was measured in years with higher value reflecting higher age.

### Dependent Variables

#### The Relative Importance of FoC

Respondents were asked to rank ten different worries in their daily lives, from the most worrisome (1) to the least worrisome (10), i.e. higher scores indicate the issue being ranked as a lower worry compared to the others. The ten listed worries included: Worry about own health, Illness of loved one, Mental health (e.g., memory issues), Death of a loved one, Becoming physically weak (e.g., fear of falling), Becoming dependent, Social isolation, Fear of Crime, Climate change and Own financial situation. In the correlation analysis (see Analytic Strategy below), participants'rank order of FoC was used as the outcome variable. To facilitate interpretation, the item was reversed so that a higher score reflects a greater level of worry.

#### The Affective (A) Aspect of FoC

Was assessed through a scale measuring the frequency of fear. This scale included seven items, each beginning with the prompt'During the past year, have you been worried about becoming a victim of…'followed by various types of crime: break-ins, car theft, online fraud, physical assault, robbery, sexual assault, and crimes committed by someone with access to one’s apartment. Respondents rated their fear of each crime on a 5-point Likert scale, from 1 (Very often) to 5 (Very seldom). The option'Never'could also be chosen but was coded as missing. To analyze the responses, the Affective FoC scale (α = 0.62) was computed by recoding the items such that responses of 0 to 2 were designated as 0 (indicating feeling safe), while responses of 3 to 4 were coded as 1 (indicating feeling unsafe), creating dichotomous variables for each item. Subsequently, the total score for the Affective FoC index was calculated as the sum of these dichotomous variables. Respondents needed to provide valid ratings for at least four of the seven items for their scores to be included in the analysis. Following this, the total score was recoded so that scores of 0 remained as 0, while scores ranging from 1 to 5 were changed to 1. This recoding resulted in a dichotomized affective FoC variable, where 0 indicated feeling safe and 1 indicated feeling unsafe.

#### The Behavioral (B) Aspect of FoC

Which reflects precautionary behavior, was assessed using two items: ‘During the past year, have you chosen a different route or mode of transportation due to fear of becoming a victim of a crime?’ and During the past year, have you avoided participating in activities, such as taking walks, going to the movies, or meeting people, due to fear of becoming a victim of crime?’ Participants rated their precautionary behavior on a 5-point Likert scale, from 1 (Very often) to 5 (Very seldom). The option ´Never´ could also be chosen but was coded as missing. To analyze the responses, the variables were recoded such that responses ranging from 0 to 2 were designated as 0 (indicating no precautionary behavior), while responses of 3 to 4 were coded as 1 (indicating precautionary behavior), creating dichotomous variables for each item. Specifically, responses were recoded using the following criteria: scores of 0, 1, or 2 remained as 0, while scores of 3 or 4 were recoded to 1. The mean score for the Behavioral FoC scale was computed based on valid responses from participants who answered at least one of the two items. The Spearman correlation between the two items yielded a reliability measure of 0.52**, as Cronbach’s alpha is not suitable for only two items. Following this, the total score for the Behavioral FoC index was calculated as the sum of the dichotomous variables. Finally, the total score was recoded so that scores of 0 remained as 0, while scores of 1 and 2 were recoded to 1, resulting in the dichotomized behavioral FoC variable.

#### The Cognitive (C) Aspect of FoC

Which measures risk of victimization, was assessed using seven items. Participants rated their agreement with the statement, ‘How likely do you think it is that you will become a victim of [specific crime] during the next 12 months?’ The items covered the same offenses as those used in the Affective FoC scale and were rated similarly on a 5-point Likert scale from 1 (Very unlikely) to 5 (Very likely). Respondents who expressed some level of worry about a specific crime on the Affective FoC scale were also asked to rate their perceived risk of that crime on the Cognitive FoC scale. Respondents who did not express worry about a specific crime did not answer the related Cognitive FoC item, leading to a lower number of valid ratings for this scale (n = 84). The Cognitive FoC scale (α = 0.47) was calculated with responses that were first dichotomized: ratings of 0 to 2 were designated as 0 (indicating low perceived risk), while ratings of 3 to 5 were recoded as 1 (indicating higher perceived risk). This created dichotomous variables for each item. Subsequently, the total score for the Cognitive FoC index was derived from the sum of these dichotomous variables. Finally, the total score was recoded such that scores of 0 remained as 0, while scores of 1 and higher were recoded to 1, resulting in the dichotomized Cognitive FoC variable. While the study originally aimed to present findings for all four measures, the cognitive aspect will not be reported due to the previously mentioned data-related issues.

### Independent Variables

The present study examined six theoretically important factors that could explain FoC in late life: three dimensions of frailty (i.e., Daily activities, Health problems, and Psychosocial functioning), Trust in the neighborhood, Perception of rising crime, and News consumption. Age and gender of the respondents were also included in the analyses. The three different dimensions of frailty were assessed using the Groningen Frailty Indicator (GFI; Steverink, 2001), a multidimensional screening tool comprising 15 items that evaluate physical, cognitive, social, and psychosocial dimensions of frailty. In this study, three dimensions of frailty were analyzed based on the GFI's previously established three-factor structure: Daily Activities, Health Problems, and Psychosocial Functioning (Bielderman et al., 2013). Higher subscale scores indicate greater levels of frailty (Schuurmans et al., 2004). For detailed information on the GFI coding procedures, see Schuurmans et al. (2004).

#### The Daily Activities

Subscale assessed the ability to carry out daily activities unassisted through four items (i.e., shopping, walking outdoors, dressing/undressing, and going to the toilet). Items were rated Yes (Independent) or No (Dependent), coded 0 and 1 respectively. The ratings for these four items were then summed up to create the Daily Activities subscale (α = 0.66). Higher scores indicate greater levels of frailty (daily activities).

#### The Health Problems

Subscale was assessed through six items (e.g., overall physical condition, visual or hearing impairments, and memory functioning). One item (overall physical condition) was rated on a scale of 1 (Very poor) to 10 (Very good), two items were coded 1 (Very problematic), 2 (Somewhat problematic) and 3 (Not at all problematic), and three items were coded 0 (No), 1 (Sometimes) and 3 (Yes). Some included variables were reversed to have higher values represent higher levels of perceived health problems. All variables were then dummy coded 0 (no/few perceived problems) and 1 (perceived health problems) and subsequently summed up to create the Health Problems subscale (α = 0.38). Higher scores indicate greater levels of frailty (health problems).

#### The Psychosocial Functioning

Subscale assessed the presence of psychosocial ailments (e.g., missing people around them, feeling abandoned, or downhearted or sad) through five items. All items were coded 0 (No), 1 (Sometimes) and 2 (Yes). Included items were dummy coded into 0 (No) and 1 (Sometimes/Yes) and then summed up to create the Psychosocial Functioning subscale (α = 0.78). Higher scores indicate greater levels of frailty (psychosocial functioning).

#### Trust in Neighborhood

Participants rated their trust in their neighborhood through one item, “How much do you trust people in your neighborhood?”. The item was rated on a four-point Likert scale where 1 (Not at all), 2 (To a small extent), 3 (To some extent), and 4 (To a large extent). A fifth response alternative was added, 5 (I do not know), which was coded as missing data before conducting the analyses. Higher scores indicate greater levels of trust in the neighborhood.

#### Perception of Crime Increasing

Participants rated their agreement to one item, i.e., “Overall, do you think that crime in Sweden has increased, decreased or remained unchanged in the last three years?”. The responses ranged from 1 (Decreased significantly) to 5 (Increased significantly). A fifth response alternative was added, 5 (I do not know), which was coded as missing data before conducting the analyses. Higher scores indicate greater levels of perceptions of increasing crime.

#### News Consumption

Participants rated the following item, “How often do you consume news, for example reading the newspaper, watching television or listening to the radio”. The item was rated on a five-point Likert scale, ranging from 1 (Multiple times a day) to 5 (I do not follow the news). The response scale for this variable was reversed before including it in the analyses, meaning higher scores reflect more extensive news consumption.

### Analytic Strategy

First, descriptive data on the relative importance of FoC and the ABC aspects of FoC were examined. Next, Spearman correlations were calculated for all study variables. The study initially intended to analyze all three aspects of Fear of Crime (FoC), namely affective, behavioral, and cognitive. The C component of FoC was excluded from the analysis due to a high proportion of missing data. This substantial number of missing values raised concerns about the reliability and generalizability of the results associated with this variable. To maintain the integrity of the analysis and ensure valid conclusions, the exclusion of this component was deemed necessary. Subsequently, two separate logistic regressions were performed to identify individual and contextual factors that independently associate with the two remaining FoC scales (Affective and Behavioral). The same predictors—gender, age, three frailty dimensions (Daily Activities, Health Problems, and Psychosocial Functioning), Trust in Neighborhood, Belief in Crime Increase, and News Consumption—were applied to the Affective FoC and the Behavioral FoC scales. It is important to note that the “Daily Activities” predictor was omitted from the Behavioral FoC analysis due to an extreme coefficient and a large standard error, which suggest potential instability in the estimates. Effect estimates will include odds ratios (ORs) for the logistic models, providing interpretable measures of effect size. Additionally, marginal effects will be calculated at the mean predicted probabilities, specifically, 0.389 for affective FoC and 0.134 for behavioral FoC, to facilitate interpretation of the magnitude of associations between independent variables and the outcomes. No issues with multicollinearity were found in any of the analyses, as the Variance Inflation Factor (VIF) values were all well below 5 (Craney & Surles, 2002). Hosmer–Lemeshow Tests showed p-values greater than 0.05 suggesting a good fit between the model and the observed data. The classification tables ranged from 61–87 percent correct. Residual diagnostics and influence measures (Cook's distance and leverage) were examined to ensure the stability of the models, and no influential outliers were identified. The highest Cook's D was below 1, and the maximum leverage value was 0.21, indicating that no individual observations exerted undue influence on the model results. For more information regarding outlier see Table 4 in the supplementary material. All analyses were conducted using IBM SPSS 28.

## Results

### How do Older Adults Rank FoC Compared to Other Common Fears and Worries in Late Life?

See Table 1, for the respondents’ ranking of different life worries for the total sample, as well as for women and men separately. Lower numbers indicate a higher ranking, meaning that the worry in question is viewed as more worrisome than the others, with 1 representing the greatest worry and 10 the least. FoC ranks near the lower end of the worry scale, at 8 out of 10, suggesting it is less worrisome compared to other common life worries among older adults. Only worries about climate change (ranked 9 th) and personal finances (ranked 10 th) are considered less worrisome than FoC. The top-ranked worry is one's own health, which receives a ranking of 1. This is followed by worries about the illness or death of a loved one and the deterioration of one's own mental health, such as cognitive memory difficulties. These results are consistent across the total group of participants and by gender, with only minor variations in rankings (not statistically verified).

**Table 1:** How does FoC rank compared to other common fears and worries in late life?

Worries about	Females (*n* = 192)		Males (*n* = 144)		Total (*n* = 336)	
	*M*	Rank	*M*	Rank	*M*	Rank
Own health	3.32	1	3.34	1	3.34	1
Illness of loved one	3.98	2	3.94	2	3.97	2
Mental health (e.g. memory issues)	4.13	3	4.85	4	4.43	3
Death of loved one	4.85	4	4.57	3	4.73	4
Becoming physically weak (e.g. fear of falling)	5.02	5	5.29	6	5.13	5
Becoming dependent	5.27	6	5.13	5	5.21	6
Social isolation	5.78	7	5.83	7	5.8	7
Fear of crime	7.01	8	6.56	8	6.82	8
Climate change	7.27	9	7.52	9	7.39	9
Own financial situation	7.7	10	7.65	10	7.68	10

Rank order from most concerning (1) to least concerning (10) issues. Means and rankings (1–10), presented by gender and for total group

### Individual and Contextual Factors Associated with the Relative Importance of FoC, and the ABC Aspects

Spearman correlation coefficients were calculated to examine associations between the study variables (see Table 2). Regarding the relative importance of FoC, the analysis shows that it is positively associated with higher perceptions of crime increasing (*rs* = 0.207, *p* < 0.001), indicating that individuals who perceive crime as rising tend to view FoC as more important. It is negatively associated with trust in the neighborhood (*rs* = −0.160, *p* < 0.01) and media consumption (*rs* = −0.110, *p* < 0.05), suggesting that individuals with lower trust in their community and less extensive media exposure perceive FoC as more significant. These findings imply that older adults who believe crime is increasing, trust their community less, and consume less media tend to prioritize fear of crime more highly.

**Table 2:** Zero-order (Spearman) correlations between all study variables

	1	2	3	4	5	6	7	8	9	10	11	12
1. Age	–											
2. Gender	-.051	–										
3. Daily activities	-.219**	-.035	–									
4. Health problems	.303**	.155**	.263**	–								
5. Psychosocial functioning	0.077	-.108	.187**	.303**	–							
6. Perception crime increasing	0.085	-.061	.059	.015	.125*	–						
7. Trust in neighborhood	.123*	.058	.064	.038	-.104	-.120*	–					
8. News consumption	-.045	.061	-.178**	-.147**	-.176**	-.195**	.125*	–				
9. Relative fear of crime	-.072	.087	-.007	-.038	.100	.207***	-.160**	−110*	–			
10. Affective fear of crime	-.032	-.049	**-**.074	.118*	.283**	.195**	-.264***	-.058	.265***	–		
11. Cognitive fear of crime	-.084	.034	-.068	-.028	.012	.211*	-.271**	.040	.219*	.172	–	
12. Behavioral fear of crime	-.006	-.066	**-.**081	-.028	.190**	.187**	-.223**	-.078	.111	.385**	.124	–

*p <.05 **p <.01 ***p <.001

Additionally, higher levels of the ABC aspects of FoC are linked to a lower trust in the neighborhood and a stronger belief that crime is increasing. Beyond these, other variables related to individual vulnerability and psychosocial functioning were examined. We found that higher levels of the ABC aspects of FoC are linked to lower trust in the neighborhood and a stronger belief that crime is increasing and that individuals with more frailty in daily activities tend to report lower levels of fear and engage in fewer precautionary behaviors, whereas those with more psychosocial problems report higher fear and are more likely to take precautions. Importantly, all FoC aspects (affective, behavioral, and cognitive) are positively correlated with each other, indicating that higher levels of one component are associated with higher levels of the others. Individuals with more frailty in daily activities tend to report lower levels of fear and engage in fewer precautionary behaviors, while those with more problems in psychosocial functioning report higher levels of fear and are more likely to take precautions. Additionally, all FoC aspects (ABC) are positively correlated with each other, meaning higher levels of one component are associated with higher levels of the others.

Table 3 presents the results from the two logistic regression analyses, forecasting the following outcomes: the Affective and the Behavioral aspects of FoC. The same regression model was applied to both outcomes. For the Affective scale, all predictors were included: demographic factors (age and gender), dimensions of frailty (Daily Activities, Health Problems, and Psychosocial Functioning), trust in one’s neighborhood, perception of crime as increasing, and news consumption. For the Behavioral scale, all predictors were similarly applied, with the exception of Daily Activities, which was omitted from the analysis.

**Table 3:** Logistic regression analyses testing the predictive ability of demographical factors, frailty dimensions, trust in neighborhood, crime increase belief, and news consumption for the AB components of FoC

Affective fear of crime (*n* = 260)						Behavioral fear of crime (*n* = 262)				
	b	*SE* (b)	Marginal effect	*OR*	(95% CI)	b	*SE*(b)	Marginal effects	*OR*	(95% CI)
Constant	-.19	.30		.83	(.46, 1.49)	.03	.43		1.25	(.49, 2.37)
Age	-.04	.02	−0.01	0.97	(-.92, 1.01)	-.02	.03	0.00	0.98	(-.92, 1.04)
Gender	-.19	.30	−0.05	0.83	(-.46, 1.49)	.03	.43	−0.00	1.03	(-.45, 2.38)
Daily activities	-.70	.47	−0.11	0.50	(-.20, 1.24)	-	-	-	-	-
Health Problems	.28*	.13	0.07	1.33	(1.03, 1.71)	.01	.17	0.00	1.01	(-.72, 1.42)
Psychosocial Functioning	.26**	.09	0.06	1.30	(1.09, 1.54)	.33**	.12	0.04	1.39	(1.09, 1.76)
Trust in neighborhood	-.87***	.22	−0.17	0.42	(-.27, -.65)	−1.00***	.28	−0.12	0.37	(.21,.63)
Crime increase belief	.51**	.19	0.12	1.67	(1.15, 2.39)	.56	.30	0.07	1.76	(.98, 3.14)
News consumption	.10	.26	0.02	1.10	.(-.66, 1.85)	-.09	.36	−0.00	0.92	(-.46, 1.85)
Nagelkerke R^2^	.26					.22				

b = Unstandardized coefficients. *OR* = Odds Ratio. *SE* = Standard Error. *CI* = Confidence Interval. The marginal effects are calculated at the mean predicted probability, (mean_pred) of 0.389 for affective fear of crime, and 0.134 for behavioral fear of crime. Nagelkerke R-squared is a pseudo-R^2^ measure that adjusts the Cox and Snell R^2^ to a scale between 0 and 1, thereby facilitating the interpretation of model fit. *p <.05, **p <.01, ***p <.001

For affective FoC, higher trust in one’s neighborhood was the strongest predictor and linked to lower levels of affective fear. Conversely, greater health problems, poorer psychosocial functioning, and the belief that crime is increasing were associated with higher levels of affective fear indicating that these factors contribute to heightened affective fear.

For behavioral FoC, higher trust in the neighborhood was also the strongest predictor and associated with lower behavioral fear. This suggests that those with greater trust in their neighborhood are less likely to change their behavior due to FoC. In contrast, higher frailty in its psychosocial dimension was significantly linked to higher behavioral fear, indicating that individuals with more psychosocial problems are more likely to change their behavior because of FoC.

## Discussion

The current study aimed to investigate FoC among older adults and to identify potential explanatory factors. Our main results revealed that, contrary to the common characterization of older adults as a generally fearful group in relation to crime, their primary worries are more centered on health problems, social isolation, and other personal vulnerabilities rather than FoC. This finding is particularly important for several reasons. First, it challenges longstanding stereotypes that depict older adults as universally fearful and vulnerable in the context of crime. Such stereotypes can lead to misguided policies and interventions that do not adequately address the real concerns faced by this demographic. Moreover, these results are consistent with prior research on older populations (Greve, 1998; Kuin & Spaans, 2017; Lenze & Wetherell, 2011) and highlight the need to reassess the overemphasis on FoC in older adults. Recognizing that health and social well-being are the primary sources of worry can lead to a more nuanced understanding of the challenges older adults face. It is imperative that we shift our focus away from viewing older adults solely through the lens of FoC and instead prioritize an understanding of their actual worries. This shift can inform not only public perception but also community planning and resource allocation. By addressing health-related concerns and promoting social connectivity, we can enhance the quality of life for older adults, ultimately resulting in more tailored and effective support systems.

Although FoC is not a major worry for the older adults in the current study, other research indicates they are more likely to stay home due to fear compared to younger individuals (Sacco & Nakhaie, 2001). In line with this, our correlation analysis revealed significant relationships between the relative importance of FoC and contextual factors in older adults. Those who believe that crime is rising in society tend to view FoC as more important. This finding aligns with earlier research, which suggests that heightened worry about crime can amplify the perceived importance of FoC, as in a “fear of crime loop” (Altheide, 2002, 2006; Heber, 2009). Such worries are consistent with previous studies showing that perceptions of safety are closely related to actual crime rates and public views on crime trends (Brunton-Smith & Sturgis, 2011; Doran & Burgess, 2012; Hale, 1996; Jackson, 2006). Furthermore, older adults who have higher trust in their neighborhood (consistent with the general population in Camacho Doyle et al., 2022) viewed FoC as less important. This suggests that a greater sense of safety and trust might mitigate the perceived threat of crime. Conversely, although some previous research indicates that news consumption is generally associated with higher FoC (Hollis et al., 2017; Näsi et al., 2021; Smolej, 2011; Altheide, 2006), our study did not demonstrate such an association. This might suggest that this relationship may not be as strong in older populations.

Furthermore, individuals with health problems (e.g., poor physical condition, visual or hearing impairments, memory issues), those with lower psychosocial functioning, and those who believe that crime is increasing experience greater affective fear. This supports the idea that physical and mental health problems (Bazargan, 1994; Hanslmaier et al., 2018; Olofsson et al., 2012), as well as perceptions of rising crime trends (Brantingham & Brantingham, 1995), may elevate general anxiety and make crime worries more pronounced, thus amplifying affective responses to crime. These findings support the vulnerability perspective on fear of crime in advanced age, highlighting that both psychical and psychosocial health problems are linked with higher worry about crime. Moreover, these findings suggest that both rationalist and symbolic paradigms play a role in explaining fear of crime in older adults (De Donder et al., 2009; Elchardus et al., 2008). Although starting from different assumptions, these two paradigms might be not completely mutually exclusive but rather complimenting each other in acknowledging the importance of both physical health (rationalist) and mental health (symbolic) in fear of crime response.

Trust in the neighborhood emerged as a key contextual factor, uniquely associated with both affective and behavioral FoC. Trust in the neighborhood was the strongest predictor at both the contextual and individual level for both concepts. Social trust plays a crucial role in reducing FoC by enhancing informal social control and encouraging community intervention (Farrall et al., 2009). Our findings indicate that for older adults, higher trust in their neighborhood is linked to reduced FoC, underscoring the significance of a supportive community environment. Building strong social ties and fostering collective efficacy (Brunton-Smith & Sturgis, 2011; Brunton-Smith et al., 2014; Camacho Doyle et al., 2022; Markowitz et al., 2001) through community events, neighborhood watch programs, and improved communication with local authorities can create a safer environment for older adults. Furthermore, older adults who trust their neighborhood are less likely to modify their behavior out of fear, suggesting that a strong sense of community stability can mitigate fear-driven actions and enhance overall well-being. 

Furthermore, higher frailty in its psychosocial dimension is associated with more cautious behavior. Thus, older individuals with more psychosocial problems, such as for instance more anxiety and depressive feelings, tend to take more behavioral precautions. However, physical health problems were not associated with the behavior aspect of FoC. This finding is in line with the symbolic paradigm in explaining FoC (De Donder et al., 2009; Elchardus et al., 2008). Specifically, we found that it is not the direct inability to withstand crime (indicated by diminished physical health and strength) which is linked to more behavioral changes, but rather the general diminished psychosocial resilience which is associated with such changes. Moreover, it adds an important nuance to the vulnerability explanation of fear in older age, highlighting that it is not physical, but rather psychological vulnerability which is associated with taking behavioral precautions and avoiding behavior because of FoC. This is especially important given that older adults tend to display more behavioral FoC than other FoC aspects, compared to younger adults (Greve et al., 2018; Kappes et al., 2013). Thus, our results suggest that aspects of both physical and psychosocial health should be included in future research on FoC in older adults.

Overall, these findings highlight the complex interplay between societal trust, perceptions of crime, and personal health in shaping FoC among older adults. While we observed differences in how explanatory factors relate to affective and behavioral FoC, fewer hypothesized predictors were significantly associated with behavioral FoC compared to its affective component. Future research should explore additional individual and contextual factors that influence the tendency to take behavioral precautions among older populations. The results also suggest that while a higher level of societal trust is generally linked to less importance and experience of FoC, a belief in increasing crime intensifies these factors. The findings also show that different dimensions of frailty are differentially related to the FoC aspects: problems in psychosocial functioning are linked to both affective and behavioral FoC, whereas health problems are linked to affective FoC only. This suggests that a nuanced approach to various aspects of health and frailty in older adulthood is beneficial for future research on FoC in older adults. The results also suggest that older adults’ perception of their environment significantly relates to their feelings and reactions toward crime.

### Strengths and Limitations

The measures used in the current study positioned FoC within the context of other age-relevant concerns, as documented in gerontological literature. Additionally, the study employed measures of frailty dimensions, which offer more nuanced and relevant indicators of later life health compared to general health questionnaires. This approach allowed for the disentanglement of the roles of physical and mental health in aspects of FoC. The findings provide new insights to theorizing FoC in older age from the vulnerability and symbolic paradigm perspectives. Thus, the study makes a unique contribution to understanding the FoC in the context of ageing.

However, the study’s limitations should be mentioned. The study's use of cross-sectional survey data limits our ability to determine causality. This means we can identify relationships between FoC, aspects of frailty and the contextual factors, but we cannot conclude that one factor directly causes changes in another. The sample was restricted to senior apartment residents, limiting generalizability to other older adults, such as homeowners or those in special-care settings such as nursing homes. For example, older adults residing in a nursing home are likely to face more health-related issues, and as such, there could be inherent differences between a sample of nursing home residents and a sample of senior apartment residents. In addition, the sample was limited to participants from one municipality only, and specific demographic and geographical factors to this municipality might hinder generalizations to older adults in other geographical areas. Participants were only those who could respond in Swedish or English. Furthermore, the frailty dimension “Health Problems” had poor internal consistency, likely due to the diverse range of health issues assessed. Additionally, the study's statistical power for analyzing cognitive FoC was limited by the sample size and number of predictors, larger samples may reveal more nuanced relationships. The age range used to signify older adults (64–106 years), is broad. Perhaps this is too wide an age range to be grouped into one sample of older adults. However, this broad age range is also a strength, allowing for a comprehensive examination of meaning across the ageing spectrum.

A noteworthy limitation of this study is the exclusion of the Cognitive (C) component of the Fear of Crime (FoC) scale. This decision was driven by a high proportion of missing data, which raised substantial concerns regarding the reliability and generalizability of results derived from this variable. The absence of the Cognitive component limits the comprehensiveness of our analysis and may affect the overall understanding of FoC among older adults. Future research should aim to address these data gaps to provide a more complete evaluation of all aspects of FoC. Additionally, the predictor variable"Daily Activities"was excluded from the Behavioral (B) FoC analysis due to an extreme coefficient and a large standard error, indicating potential instability in the estimates. Although a total of 260 participants provided responses, only 43 reported experiencing fear within the behavioral aspect. This limited number of fearful participants reduces the robustness of the analysis and may inflate standard errors or obscure true relationships among variables. Furthermore, it is plausible that older adults who struggle with daily functioning may inherently avoid certain activities, influencing their reported precautionary behaviors related to fear of crime. This exclusion may impact the interpretation of factors influencing behavioral responses to fear of crime. Future studies should focus on exploring these relationships within larger samples and consider the implications of low fear reporting on behavioral outcomes in this population.

## Conclusions

The current study reassesses FoC among older adults, highlighting that their primary worries often center around health issues, social isolation, and personal vulnerabilities rather than crime. By applying a relatively novel methodological approach – ranking fear of crime among other worries – the study challenges the common belief that crime is a primary fear for the older population in general. The findings suggest a need to refocus efforts on addressing those older adults for whom FoC is more relevant, such as individuals who believe crime is increasing. Additionally, it is important to consider older adults who exhibit more pronounced FoC, including those with greater psychosocial frailty, less trust in their neighborhoods, and a perception of rising crime rates. To effectively address the primary worries of older adults, prioritizing initiatives that improve healthcare access, promote physical activity, and alleviate social isolation may be more impactful than solely aiming to reduce their FoC. Interventions should therefore focus on enhancing healthcare access, encouraging physical activity, and reducing social isolation. While crime remains a concern for some older adults, enhancing community safety and trust through neighborhood watch programs and community engagement can serve to complement these broader efforts. Effective safety communications should address health and social issues alongside crime prevention to resonate with older adults'primary worries.

Taken together, this study makes a distinctive contribution to understanding fear of crime in older adults by explicitly framing it within the broader context of aging as a life phase. The innovative use of rank ordering of life worries, combined with the inclusion of frailty as an age-relevant health predictor, offers a methodological advancement in fear of crime research. Moreover, this work bridges criminological perspectives with gerontological knowledge, providing valuable insights for both fields. The findings also inform safety-promoting policies by identifying psychosocial frailty (at the individual level) and trust in the neighborhood (at the contextual level) as potential targets for interventions aimed at enhancing perceived safety among older populations.

Future research should extend the investigation of the relative importance of fear of crime across diverse living environments, such as homeowners and various care facilities, to better understand how FoC varies across different residential settings. Longitudinal studies could shed light on how crime-related worries and associated factors evolve over time. Examining various forms of social support and how neighborhood trust, media consumption, and crime perceptions interact with health and frailty longitudinally will provide deeper insights. This approach will help create more effective strategies for supporting older adults by addressing the complex relationship between FoC, health, and social factors. By acknowledging and addressing the multifaceted concerns of older adults, particularly health and social integration, we can promote a more comprehensive approach to their well-being that not only alleviates FoC but also enhances their quality of life.

## Supplementary Information

Below is the link to the electronic supplementary material.Supplementary file1 (DOCX 17 KB)

## Data Availability

The data are not made publicly available due to participant confidentiality. Requests to access the data could be sent to Karin Hellfeldt, principal investigator of the 65+ and Safe Study: karin.hellfeldt@oru.se.
