# Supplementary material for: Beyond Fear of Crime: Exploring the True Worries of Older Adults in the Context of Fear of Crime and Vulnerability in Sweden
Source: Eur J Crim Pol Res. 2025 Jun 25;32(3):797–817. doi: 10.1007/s10610-025-09631-2 (PMC13521968; doi:10.1007/s10610-025-09631-2)
Supplement: Supplementary file 1 [file 10610_2025_9631_MOESM1_ESM.docx]

Table 4. Residuals for Affective and Behavioral Fear of Crime

|  | Affective fear of crime  (n=260) |  | |  | | Behavioral fear of crime  (n=262) |  | |  | |
| --- | --- | --- | --- | --- | --- | --- | --- | --- | --- | --- |
| Case | Observed | Predicted | Residual | | ZResidual | Observed | Predicted | Residual | | ZResidual |
| 1 | 1 | 0.144 | 0.856 | | 2.443 | - | - | - | | - |
| 2 | 1 | 0.135 | 0.865 | | 2.528 | - | - | - | | - |
| 3 | 1 | 0.090 | 0.910 | | 3.177 | 1 | 0.011 | 0.989 | | 2.998 |
| 4 | 1 | 0.127 | 0.873 | | 2.625 | - | - | - | | - |
| 5 | 0 | 0.883 | -0.883 | | -2.748 | - | - | - | | - |
| 6 | - | - | - | | - | 1 | 0.066 | 0.934 | | 3.757 |
| 7 | - | - | - | | - | 1 | 0.082 | 0.918 | | 3.349 |
| 8 | - | - | - | | - | 1 | 0.071 | 0.929 | | 3.624 |
| 9 | - | - | - | | - | 1 | 0.079 | 0.921 | | 3.409 |
| 10 | - | - | - | | - | 1 | 0.126 | 0.874 | | 2.639 |
| 11 | - | - | - | | - | 1 | 0.079 | 0.921 | | 3.426 |
| 12 | - | - | - | | - | 1 | 0.131 | 0.869 | | 2.574 |
| 13 | - | - | - | | - | 1 | 0.103 | 0.897 | | 2.944 |
| 14 | - | - | - | | - | 1 | 0.055 | 0.945 | | 4.158 |

Note. Residuals represent the difference between the observed values and the predicted probabilities from the logistic regression model. ZResiduals are standardized residuals indicating how many standard deviations each residual is from the mean; values greater than ±2 are considered potential outliers. Influence measures such as Cook's distance and leverage did not indicate any influential data points, as the highest Cook's D was below 1 and the maximum leverage was 0.21. This suggests that there are no cases exerting undue influence on the model estimates. Entries marked with “-” indicate cases where no corresponding observation exists for that analysis.
